# Genetic correlations among diaphragmatic, femoral, inguinal, ventral, and umbilical hernias and identification of their candidate genes

**DOI:** 10.1097/JS9.0000000000004699

**Published:** 2026-01-07

**Authors:** Hekai Shi, Hui Ma, Qijun Li, Junliang Ma, Xiaoyu Peng, Dongchao Yang, Zhicheng Song, Yan Gu

**Affiliations:** a Department of General Surgery, Huadong Hospital, Fudan University, Shanghai, People’s Republic of China; b Department of Vascular Surgery, Huadong Hospital, Fudan University, Shanghai, People’s Republic of China; c Department of Nursing, Huadong Hospital, Fudan University, Shanghai, People’s Republic of China

**Keywords:** diaphragmatic hernia, genetic correlation, inguinal hernia, Mendelian randomization, ventral hernia

## Abstract

**Objective:**

Different subtypes of hernia exhibit significant familial aggregation. However, the genetic correlations among different hernia subtypes, as well as their shared and subtype-specific candidate genes still remain unclear.

**Methods:**

Using genome-wide association study (GWAS) data from the FinnGen consortium, we quantified genetic correlations (*r*_g_) among diaphragmatic (16 034 cases), inguinal (43 066 cases), femoral (1 660 cases), and hernia of abdominal wall (21 210 cases, including umbilical and ventral hernias) by linkage disequilibrium score regression. Mendelian randomization (MR) analyses were performed using 15 695 expression quantitative trait loci (eQTL) as exposures and hernia GWAS data as outcomes. Multiple testing was controlled using the false discovery rate (*q* < 0.05), and findings were further prioritized through Bayesian colocalization. Shared candidate genes were validated using fibroblast eQTL data from GTEx V10 and protein quantitative trait loci data from the UK Biobank.

**Results:**

Significant genetic correlations were observed across all hernia subtypes (*P* < 0.01), with the strongest correlation between ventral and umbilical hernias (*r*_g_ = 0.78), while diaphragmatic hernia exhibited weaker correlations with other subtypes (*r*_g_ = 0.26–0.48). MR analyses using blood and fibroblast-derived eQTL data identified *BMP6, MYCBPAP*, and *ZNF75A* as candidate genes shared across multiple hernia subtypes. Proteomic validation further revealed that elevated BMP6 protein levels were associated with a reduced risk for all hernia subtypes.

**Conclusion:**

This study reveals a shared genetic basis among common hernia subtypes and identifies three shared candidate genes (*BMP6, MYCBPAP*, and *ZNF75A*). Elevated BMP6 protein levels were associated with a reduced risk of multiple hernia types. These findings provide new insights into the genetic correlations and potential pathogenic mechanisms underlying hernia. Future functional studies are needed to elucidate the roles of these genes in abdominal wall tissue.

## Introduction

Hernia is a common surgical condition characterized by the abnormal protrusion of intra-abdominal contents through congenital or acquired defects, while surgical repair remains the therapeutic intervention^[1–3]^. In 2019, hernia of abdominal walls affected approximately 30 million people worldwide, and this number is projected to rise to nearly 40 million by 2030, representing a substantial public health burden^[4]^.

**HIGHLIGHTS**

- Quantified significant genetic correlations among diaphragmatic, inguinal, femoral, ventral, and umbilical hernias.
- Revealed both shared and subtype-specific candidate genes for multiple hernia subtypes.
- Multilayer Mendelian randomization identifies *BMP6, MYCBPAP*, and *ZNF75A* as shared candidate genes across multiple hernia subtypes.
- Proteomic analyses confirm that elevated BMP6 protein levels confer protection against all hernia subtypes.

Hernia occurrence and recurrence show strong familial clustering and are closely linked to collagen metabolism disorders^[5–7]^. Genome-wide association study (GWAS) has identified candidate genetic loci conferring susceptibility to hernia of the abdominal wall^[8–10]^. However, GWAS identify only statistical associations and cannot establish causal relationships. Mendelian randomization (MR) uses genetic variants as instrumental variables (IVs) to support causal inference between exposures and outcomes, thereby minimizing confounding inherent in observational studies^[11]^.

To date, few studies have used MR to systematically assess the causal roles of specific genes in different hernia subtypes. In this study, we used Linkage disequilibrium score regression (LDSC) to quantify genetic correlations across multiple hernia subtypes, including diaphragmatic, inguinal, femoral, umbilical, ventral, and hernia of abdominal wall (a composite phenotype comprising ventral, umbilical, and other hernia of abdominal wall). We then conducted two-sample MR analyses using single-nucleotide polymorphisms (SNPs) associated with expression quantitative trait loci (eQTL) in whole blood and connective tissue fibroblasts as IVs to identify both subtype-specific and shared candidate genes^[12]^. The primary aim of this study was to identify genetic associations across multiple hernia subtypes and to determine both shared and subtype-specific candidate genes. We hypothesized that hernia subtypes share a genetic basis, with certain genes influencing susceptibility across multiple subtypes. This study adhered to the TITAN guidelines, and no artificial intelligence tools were utilized for data analysis or manuscript preparation^[13]^.

## Methods

### Data sources

**Figure 1.**
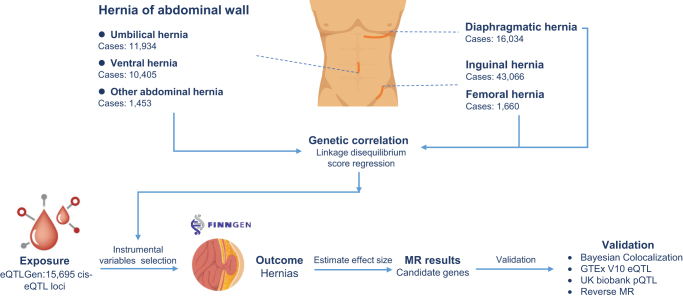
Flow chart of the study design. Genetic correlations among multiple hernia subtypes were first assessed using linkage disequilibrium score regression. Mendelian randomization analyses were then performed using eQTLs from the eQTLGen consortium as exposures and various hernia subtypes as outcomes. Candidate hernia-associated genes were identified via Bayesian colocalization and further validated using pQTLs from the UK Biobank and eQTLs from GTEx V10. eQTL, expression quantitative trait loci; pQTL, protein quantitative trait loci; MR, Mendelian randomization.

The study design is summarized in Figure 1. GWAS summary statistics for hernia subtypes were obtained from the FinnGen project (Release 12), a large-scale biobank integrating genetic data with nationwide electronic health records from the Finnish population^[14]^. Disease endpoints in FinnGen are defined using comprehensive national registries, including the Hospital Discharge and Causes of Death Registers. Phenotype definitions for each hernia subtype were based on official FinnGen phenocodes with corresponding ICD-10, ICD-9, and ICD-8 mappings, all publicly accessible (https://r12.finngen.fi/). Table 1 details the phenocodes, sample sizes, and specific ICD codes for all analyzed subtypes. This resource provides a robust foundation for investigating the genetic correlations among diaphragmatic, inguinal, femoral, and hernia of abdominal wall. In the FinnGen GWAS data used for our analyses, sex, age, and the first ten genetic principal components were included as covariates to control for population stratification and demographic effects (https://docs.finngen.fi/finngen-data-specifics/red-library-data-individual-level-data/genotype-data/types-of-genotype-files-available/covariate-file). Therefore, potential confounding effects of sex and ancestry were already controlled in the association models.

**Table 1:** Definitions and sample sizes of hernia subtypes in the FinnGen cohort.

Hernia types	Phenocode in FinnGen	ICD codes or description (hospital discharge or cause of death)	Sample size
Diaphragmatic hernia	K11_DIAHER	ICD-10 – K44	16 034 cases
		ICD-9 – 5513|5523|5533	
			426 666 controls
		ICD-8 – 5513	
Femoral hernia	K11_FEMHER	ICD-10 – K41	1 660 cases
		ICD-9 – 5510|552A|5530	
			426 666 controls
		ICD-8 – 5510	
Inguinal hernia	K11_HERING	ICD-10 – K40	43 066 cases
		ICD-9 – 550	426 666 controls
		ICD-8 – 550	
Ventral hernia	K11_VENTHER	ICD-10 – K43	10 405 cases
		ICD-9 – 5512|5522|5532	
			426 666 controls
		ICD-8 – 5512	
Umbilical hernia	K11_UMBHER	ICD-10 – K42	11 934 cases
		ICD-9 – 5511|5521|5531	
			426 666 controls
		ICD-8 – 5511	
Other and unspecified abdominal hernia (obturator, lumbar hernia, etc.)	K11_OTHABDHER	ICD-10 – K45, K46	1453 cases
		ICD-9 – 551[8-9]|552[8-9]|553[8-9]	
			426 666 controls
		ICD-8 – 551[8-9]	
Hernia of abdominal wall	ABDOM_HERNIA	K11_UMBHER	21 210 cases^a^
		K11_VENTHER	479 138 controls
		K11_OTHABDHER	


ICD, International Classification of Diseases codes.

^a^ The total case count after deduplication among ventral hernia, umbilical, and Other and unspecified abdominal hernia.

Whole-blood eQTL data were obtained from the eQTLGen Consortium, which identified 15 695 cis-eQTL loci based on transcriptome sequencing of 31 684 individuals^[15]^. As the largest and most statistically robust whole-blood eQTL dataset currently available, eQTLGen provides reliable instrumental variables, enhancing the accuracy of causal inference in MR analyses^[15]^.

For tissue-specific validation, we used fibroblast eQTL data from skin samples in the GTEx Project V10. GTEx collected up to 54 nondiseased tissue types from nearly 1000 donors, all of whom underwent whole-genome sequencing and bulk RNA sequencing to evaluate the effects of genetic variation on gene expression^[16]^. Protein quantitative trait loci (pQTL) data were obtained from the UK Biobank Pharma Proteomics Project, which identified 1812 pQTL loci suitable for MR analysis through plasma proteome profiling of 54 219 participants^[17]^.

All data used in this study were obtained from public databases. As the original GWAS was conducted with ethics committee approval and all data were anonymized, no additional ethical approval was required for this study.

### Genetic correlation analysis

LDSC uses GWAS summary statistics to estimate heritability (*h*^2^) and genetic correlations. When a phenotype has nonsignificant heritability, genetic correlation estimates may be unreliable^[18,19]^. Therefore, only hernia subtypes with significant SNP-based heritability (*P* < 0.05) were included in subsequent genetic correlation analyses. LDSC estimates the degree of shared genetic correlation between traits by calculating the genetic correlation coefficient (*r*_g_) from GWAS summary statistics^[20]^. Using GWAS summary statistics for both hernia types, we estimated the genetic correlation using linkage disequilibrium (LD) scores derived from the 1000 Genomes Project European reference panel. The *r*_g_ value ranges from −1 to 1, with a positive correlation indicating concordant genetic effects and potential overlap in the underlying causal variants between the two hernia phenotypes.

### MR analysis

MR used SNPs as IVs to estimate the causal effect of eQTLGen-derived gene expression on hernia subtypes. SNPs showing genome-wide significant associations with gene expression (*P* < 5 × 10^−8^) were selected from eQTL summary statistics as candidate IVs, and independent variants were retained after LD clumping (*r*^2^ < 0.001, window = 10 000 kb). IVs with *F*-statistics < 10 were excluded to reduce weak instrument bias. Effect estimates and standard errors (SE) for the selected IVs were extracted from the hernia GWAS datasets. The inverse-variance weighted (IVW) method served as our primary analytical approach, providing odds ratios (ORs) with 95% confidence intervals for hernia risk per one-standard-deviation increase in gene expression. This method integrates weighted effect estimates from multiple instrumental variants to deliver unbiased causal estimates under the assumption of no horizontal pleiotropy^[21]^.

To assess the robustness of the MR findings, we performed sensitivity analyses using leave-one-out analysis and MR-PRESSO for outlier detection. *P* values from the IVW analysis were adjusted for the false discovery rate (FDR), with significance defined as an FDR-corrected *P* value (*q*) < 0.05.

### Bayesian colocalization analysis

Bayesian colocalization analysis was applied to validate MR-identified genes associated with hernia risk. This method tests whether eQTL and GWAS signals share a common causal variant and estimates posterior probabilities (PP) for four hypotheses: (1) no association for either trait; (2) association with the eQTL only; (3) association with the disease only; and (4) a shared causal variant underlying both traits. A *P* value > 0.75 for hypothesis (4) indicates a high probability of colocalization, suggesting the presence of a shared causal variant within the genomic region. Bayesian colocalization analysis helps distinguish shared causal signals from associations driven by LD, thereby prioritizing candidate genes that may contribute to disease pathogenesis.

### Validation using GTEx V10 eQTL and UK Biobank pQTL data

To validate genes with shared causal variants across hernia subtypes identified by Bayesian colocalization analysis, we performed MR by integrating hernia GWAS summary statistics with fibroblast eQTL data from GTEx V10 and plasma pQTL data from the UK Biobank. The fibroblast eQTL data provided tissue-specific validation of causal effects in a connective tissue context. Additionally, we used pQTL data to investigate whether circulating protein levels encoded by the candidate genes influence hernia risk.

### Reverse MR analysis

To evaluate potential reverse causality, we conducted reverse MR analysis with hernia risk as the exposure and candidate genes shared across hernia subtypes as outcomes. This analysis aimed to determine whether the expression levels of these candidate genes were causally influenced by the risk of corresponding hernia subtypes.

### Data analysis and statistics

All analyses were performed using R (R Foundation for Statistical Computing, version 4.3.1). Key analyses and data visualization were conducted with established R packages, including TwoSampleMR, coloc, and ggplot2. A two-sided *P* value of less than 0.05 defined statistical significance for all tests.

## Results

### Genetic correlations among hernia subtypes

**Figure 2.**
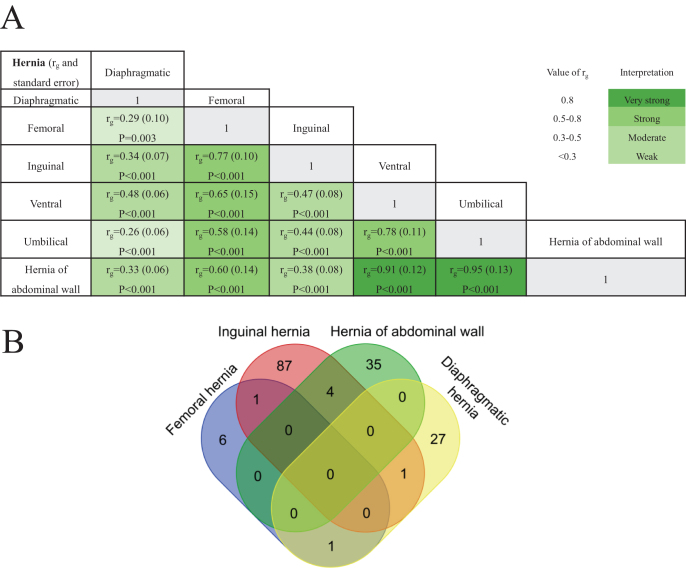
Genetic correlation and results of Mendelian randomization analysis of shared candidate genes for hernia subtypes. (A) Genetic correlation coefficients (*r*_g_) among multiple hernia subtypes and *P* value of their significance. (B) Venn diagram of the candidate genes specific to the main subtypes of hernias.

The heritability estimate for other abdominal hernias (OTHABDHER) was not statistically significant, suggesting this category constitutes a heterogeneous and poorly defined group (Supplemental Digital Content Table S1, available at: http://links.lww.com/JS9/G606). Consequently, it was excluded from subsequent genetic correlation analyses. Using LDSC analysis, we observed varying degrees of genetic correlation among most hernia subtypes (Fig. 2A). Specifically, umbilical and ventral hernias, as the two major subtypes of hernia of abdominal walls, showed a strong genetic correlation (*r*_g_ = 0.78, SE = 0.11, *P* < 0.001), indicating shared genetic influences. A similarly strong correlation was detected between inguinal and femoral hernias (*r*_g_ = 0.77, SE = 0.10, *P* < 0.001), supporting shared genetic mechanisms among groin hernias. In contrast, diaphragmatic hernia demonstrated relatively weak genetic correlations with other subtypes (*r*_g_ range: 0.26–0.48), suggesting a more distinct genetic etiology. Notably, the broader category “hernia of the abdominal wall” showed high genetic correlations with both umbilical (*r*_g_ = 0.95, SE = 0.13, *P* < 0.001) and ventral hernias (*r*_g_ = 0.91, SE = 0.12, *P* < 0.001), indicating its strong representativeness of hernia of abdominal wall subtypes.

### MR analysis results and colocalization analysis

After applying FDR correction, MR analysis identified 588 genes significantly associated with the risk of diaphragmatic hernia, 849 with inguinal hernia, 473 with umbilical hernia, 504 with ventral hernia, 589 with hernia of the abdominal wall, and 179 with femoral hernia.

We then performed Bayesian colocalization analysis on genes significantly associated with hernias. A total of 29 genes were strongly colocalized with diaphragmatic hernia, 93 with inguinal hernia, 41 with umbilical hernia, 22 with ventral hernia, 39 with hernia of the abdominal wall, and 8 with femoral hernia (Supplemental Digital Content Tables S2–S7, available at: http://links.lww.com/JS9/G606).

**Figure 3.**
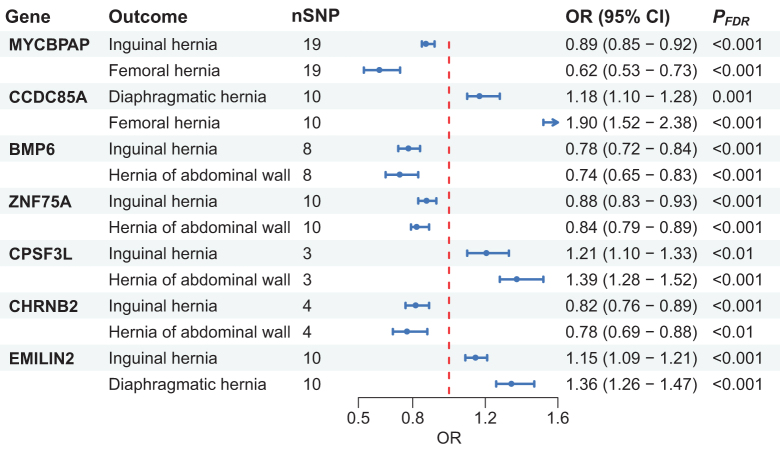
MR analysis results for shared candidate genes. Seven candidate genes associated with multiple hernia subtypes were identified through Bayesian colocalization. Notably, the risk effects of these shared genes were consistent in direction across the respective hernia types, with no conflicting effect directions observed. FDR, false discovery rate; OR, odds ratio; SNP, single-nucleotide polymorphisms.

Among the hernia subtypes, one shared candidate gene was identified between each of the following pairs: inguinal–femoral, diaphragmatic–inguinal, and diaphragmatic–femoral hernias. Additionally, four shared risk loci were found between inguinal and hernia of abdominal wall (Fig. 2B). Notably, all seven shared genes exhibited uniformly protective or detrimental effects across their associated hernia types (Fig. 3). Specifically, *MYCBPAP* was associated with a decreased risk of both inguinal and femoral hernias; *CCDC85A* increased risk for diaphragmatic and femoral hernias; *EMILIN2* elevated risk of diaphragmatic and inguinal hernias; *BMP6, ZNF75A*, and *CHRNB2* were associated with reduced risk of inguinal and hernia of abdominal walls, while *CPSF3L* was related to increased risk of both inguinal and hernia of abdominal walls.

We highlight the top 10 genes per subtype based on association strength. For diaphragmatic hernia, elevated expression of *MED24, NLRC4, GCAT*, and *AC011816.1* was protective, whereas *RP11-62H7.2, UQCC, SNX11, ZNHIT6, PSMC5*, and *EMILIN2* were associated with increased risk. For inguinal hernia, *GNL3, DALRD3, ARIH2, BMP6, LTB4R, P4HTM, MYCBPAP*, and *TMEM204* were protective, while *USP35* and *QRICH1* conferred elevated risk. For femoral hernia, *ING2, LEF1-AS1, MYCBPAP*, and *RPGRIP1* were protective, whereas *KLF11, MKNK2, AC097500.2*, and *CCDC85A* increased risk.

For umbilical hernia, six genes (*STK25, BMP6, SMAD3, C1QA, RHOC*, and *BOK*) were associated with reduced risk, and four genes (*C4orf32, PPP1R14A, NOC3L*, and *SPSB1*) were linked to increased risk. For ventral hernia, four genes (*ZNF75A, OPRL1, ANKRD65*, and *CHRNB2*) were associated with decreased risk, while six genes (*CPSF3L, PCCA, ATP11B, BSG, SLC2A5*, and *WDR1*) were linked to increased risk. In hernia of the abdominal wall, five genes (*ANKRD65, C1QA, ZNF75A, CHRNB1*, and *TYMP*) were associated with reduced risk, while five other genes (*RP11-464D20.6, DLEU1, NOC3L, SPSB1*, and *CPSF3L*) were related to increased risk. Detailed MR and sensitivity analyses for loci associated with all subtypes of hernia were provided in the Supplemental Digital Content Tables S8–S13, available at: http://links.lww.com/JS9/G606.

### GTEx V10 eQTL and UK Biobank pQTL validation

**Figure 4.**
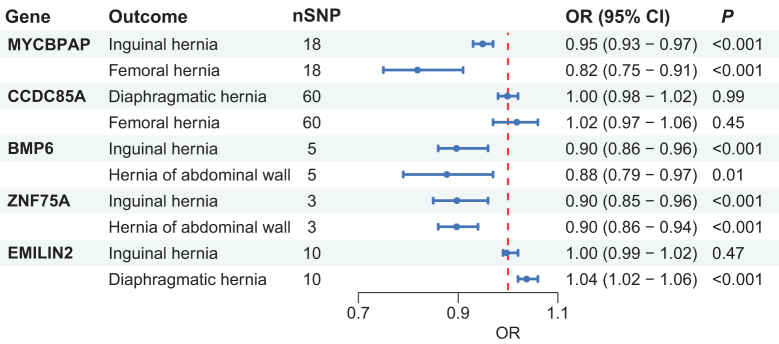
Fibroblast eQTL data from GTEx V10 further validated the causal relationships of MYCBPAP, BMP6, and ZNF75A with the risk of multiple hernia subtypes. OR, odds ratio; SNP, single-nucleotide polymorphisms.

Of the seven candidate genes identified, five had detectable eQTLs in GTEx V10 fibroblasts. Among these, seven gene-hernia pairs (70%) showed causal directions consistent with previous whole-blood eQTL analyses (Fig. 4). Exceptions included *CCDC85A* with diaphragmatic and femoral hernias and *EMILIN2* with inguinal hernia displayed inconsistent effect direction, potentially reflecting tissue-specific regulatory differences.

We further assessed protein-level associations using UK Biobank pQTL data. Because pQTL coverage was limited (1812 pQTLs versus 15 695 eQTLs), only BMP6 among the shared risk genes could be validated. MR analysis supported a causal effect of elevated circulating BMP6 protein levels on a reduced risk of multiple hernia subtypes (Supplemental Digital Content Figure S1, available at: http://links.lww.com/JS9/G605).

### Reverse MR analyses

To assess potential reverse causality, we performed reverse MR analyses to test whether hernia risk influences the expression of shared candidate genes identified by Bayesian colocalization. Of the 14 hernia-gene pairs examined, we excluded two (femoral hernia-MYCBPAP and inguinal hernia-EMILIN2) due to insufficient instrumental variables. Reverse MR indicated a bidirectional relationship between hernia of the abdominal wall and inguinal hernia risk with *CPSF3L* expression. For the remaining eight pairs, no evidence of reverse causality was detected, reinforcing the robustness of our primary findings (Supplemental Digital Content Figure S2, available at: http://links.lww.com/JS9/G605).

## Discussion

By integrating GWAS and MR analyses, this study systematically investigated the genetic correlations among diaphragmatic, inguinal, femoral, umbilical, abdominal, and ventral hernias, identifying both shared and subtype-specific candidate genes for hernia risk. Our results confirmed significant genetic correlations across the six hernia subtypes. Bayesian colocalization analysis identified seven candidate genes shared among multiple hernia subtypes. Further validation using reverse MR and fibroblast-derived eQTL data confirmed three key shared candidate genes, namely *BMP6, MYCBPAP*, and *ZNF75A*, which showed consistent causal associations across several hernia subtypes. Subsequent proteomic validation demonstrated that elevated plasma BMP6 protein levels were significantly associated with a reduced risk of all hernia subtypes. Overall, our findings establish a shared genetic basis for major hernia subtypes and offer mechanistic insights into hernia pathogenesis.

In LDSC analyses, substantial sample overlap can potentially affect the estimation of genetic correlations. In our study, some individuals were diagnosed with more than one hernia subtype, leading to a certain degree of case overlap across subtypes. According to the FinnGen statistics, except for hernia of abdominal wall, which inherently includes ventral and umbilical hernias, overlap rates between other subtypes were below 10% (Supplemental Digital Content Table S14, available at: http://links.lww.com/JS9/G606). Moreover, LDSC accounts for nongenetic covariance arising from sample overlap or population structure through its intercept. As shown by Bulik-Sullivan *et al*, mild sample overlap does not substantially bias the *r*_g_ estimate^[20,22]^. Therefore, the genetic correlation results reported in this study are considered robust.

As a member of the TGF-β superfamily, bone morphogenetic protein 6 (BMP6) activates the SMAD1/5/8 signaling pathway through receptor binding, thereby regulating fibroblast proliferation and collagen synthesis and playing a critical role in tissue repair^[23,24]^. Besides its established role in bone regeneration, the BMP6/SMAD pathway also promotes endothelial cell proliferation and neovascularization, thereby supporting tissue regeneration^[25]^. While moderate collagen deposition aids in restoring structural integrity, excessive deposition causes fibrotic scarring, compromises tissue tensile strength, and increases the risk of hernia^[26–28]^. Consistent with this, studies in transgenic male mice overexpressing aromatase have shown that elevated estrogen production in the lower abdominal muscle induces widespread muscle fibrosis and subsequent hernia formation^[29,30]^.

Topical application of BMP6 has demonstrated significant anti-fibrotic effects in localized scleroderma models, supporting its potential as a therapeutic target in fibrotic diseases^[31,32]^. Conversely, BMP6 deficiency or downregulation exacerbates renal fibrosis and cardiac fibrosis following myocardial infarction^[33,34]^. Based on our findings, we propose that elevated *BMP6* expression in inguinal and hernia of abdominal wall may reduce hernia risk by mitigating excessive fibrosis and maintaining collagen metabolic balance. This hypothesis supports the established model that chronic inflammation-induced fibrosis weakens the abdominal wall and facilitates hernia formation^[30,35]^. Previous studies have demonstrated BMP6 expression and function in skeletal muscle progenitor cells and adipogenic/mesenchymal precursors^[36,37]^. In vitro, BMP6 drives skeletal muscle progenitors toward a brown adipocyte–like phenotype, and BMP signaling is required for satellite cell maintenance and muscle mass control. Inhibition of this pathway induces muscle atrophy in mice^[38,39]^. Collectively, these findings highlight the biological relevance of BMP6 in muscle and abdominal wall physiology. The roles of *MYCBPAP* and *ZNF75A* in collagen metabolism and hernia pathogenesis are previously unrecognized and require further investigation.

The primary strength of this study lies in its integration of diverse methodologies with multitiered validation, which strengthens the findings and provides robust biological evidence. However, several aspects require further investigation. We acknowledge that the analysis integrated outcome data from the FinnGen cohort (primarily of Finnish ancestry), exposure data from the UK Biobank, and eQTL data from eQTLGen and GTEx V10, both derived largely from European-ancestry populations. Differences in ancestry across these cohorts may introduce potential bias due to population-specific allele frequencies and LD patterns^[40]^. Although the original GWAS analyses adjusted for age, sex, and the first ten genetic principal components, residual population stratification may affect the generalizability of results, particularly beyond European populations. Furthermore, while colocalization and MR analyses identified associations between specific candidate genes and hernia subtypes, the molecular mechanisms through which these genes exert their effects remain unclear. Future studies need to determine how these genes influence hernia development, using in vitro and in vivo models.

In conclusion, this study reveals a shared genetic basis among common hernia subtypes and identifies three shared candidate genes (*BMP6, MYCBPAP*, and *ZNF75A*). Elevated BMP6 protein levels were associated with a reduced risk of multiple hernia types. These findings provide new insights into the genetic correlations and potential pathogenic mechanisms underlying hernia.

## Data Availability

The data presented in this article are publicly available.

## Research registration unique identifying number

Study registration was not applicable to this work.
